# Recent advances in autoantibody-mediated pain

**DOI:** 10.1097/SPC.0000000000000820

**Published:** 2026-07-07

**Authors:** Adham Farah, John M. Dawes

**Affiliations:** Nuffield Department of Clinical Neurosciences, University of Oxford, Oxford, UK

**Keywords:** autoantibodies, autoimmune pain, dorsal root ganglion, neuroimmune mechanisms, neuropathic pain

## Abstract

**Purpose of review:**

Recent studies increasingly support a role for autoantibodies in selected chronic pain disorders. This review examines current evidence linking autoantibodies to nociceptive sensitisation and neuroimmune dysfunction across these conditions.

**Recent findings:**

Experimental and clinical evidence links autoantibodies to altered neuronal excitability, neuroimmune activation, and pain hypersensitivity, particularly within the dorsal root ganglion and spinal sensory circuits. Emerging work further suggests that pathogenicity and treatment responsiveness depend on factors beyond antibody specificity alone, including tissue injury, inflammatory context, and downstream immune signalling mechanisms.

**Summary:**

Autoimmune pain disorders appear to comprise biologically heterogeneous neuroimmune syndromes in which dorsal root ganglia and anatomically connected sensory pathways emerge as recurring sites of sensitisation. Pathogenicity depends not only on antibody specificity but also on tissue context and downstream neuroimmune mechanisms, supporting the development of more targeted and mechanism-based therapeutic approaches.

## INTRODUCTION

Pain remains a major clinical challenge, characterised by limited treatment efficacy and an incomplete understanding of the mechanisms that sustain chronic symptoms. While neuroimmune interactions are recognised contributors to pain [1], existing models do not fully explain the variability and persistence of chronic pain conditions.

Accumulating evidence now implicates autoantibodies (Ab) as active mediators of pathological pain rather than solely biomarkers of immune activation [2]. Using experimental models, patient-derived autoantibodies have been shown to alter neuronal excitability, sensory processing, and neuroimmune signalling [3,4,5^▪^^▪^,^6^], while plasma exchange or immunotherapies, such as intravenous immunoglobulin (IVIG), can alleviate pain in subsets of patients [7,8,9^▪^]. However, therapeutic response remains variable, indicating that autoantibody-mediated pain is mechanistically heterogeneous and strongly influenced by disease context [10].

Autoantibody-associated pain has been described across diverse pain conditions [2_,_^11^], with recent studies progressing our understanding of autoantibody contributions in particular conditions such as autoimmune neurological disorders, peripheral sensory neuropathies, fibromyalgia (FMS), and injury-associated pain states. Despite substantial differences in antigen specificity and clinical phenotype, these disorders repeatedly involve disruption of sensory processing associated with diverse antibody-mediated effects on sensory neurons, neuroimmune interfaces, and nociceptive circuits.

Here, we provide an update on selected autoantibody-mediated pain conditions, in which recent studies have substantially advanced mechanistic understanding, highlighting insights into sensory dysfunction, anatomical sites of autoantibody action, context-dependent pathogenicity, and the clinical implications for mechanism-based therapies.

**KEY POINTS**

- Recent studies increasingly support pathogenic roles for autoantibodies in subsets of chronic pain disorders through effects on sensory neurons and neuroimmune signalling pathways.
- Across multiple conditions, dorsal root ganglia and anatomically connected sensory pathways emerge as important sites of autoantibody-associated sensitisation.
- Autoimmune pain mechanisms are mechanistically heterogeneous and may involve neuronal hyperexcitability, Fc receptor signalling, glial activation, or inflammatory amplification.
- Tissue injury, inflammatory context, and local immune signalling critically influence whether autoantibodies become pronociceptive and sustain chronic pain.
- Despite substantial mechanistic advances, limited controlled clinical evidence remains a major challenge in translating autoantibody-associated pain mechanisms into patient stratification and targeted treatment strategies.

## CASPR2 AND LGI1 AUTOIMMUNITY

Autoantibodies targeting proteins of the voltage-gated potassium channel (VGKC) complex, although rare, remain among the best-studied examples of antibody-mediated pain. Rather than targeting potassium channels directly, these antibodies disrupt associated proteins regulating neuronal excitability, particularly contactin-associated protein-like 2 (CASPR2) and leucine-rich glioma-inactivated 1 (LGI1).

Pain, particularly neuropathic pain, is increasingly recognised in both CASPR2 and LGI1 autoimmunity and occurs alongside other common features such as seizures, cognitive dysfunction, and muscle hyperexcitability [7_,_^9^^▪^]. Earlier cohort studies identified pain as a frequent manifestation of CASPR2 autoimmunity, occurring in approximately 30–50% of patients and more commonly than in LGI1-associated disease (52 vs. 19%) [7_,_^10^_,_^12^_,_^13^]. More recent CASPR2-specific cohorts further reported chronic pain in 36% of patients, often severe and sometimes the predominant clinical manifestation [13]. The emerging phenotype predominantly involves distal burning neuropathic pain, although widespread pain and small-fibre dysfunction are also recognised [9^▪^,^13^]. A recent systematic review of 216 CASPR2-Ab patients with neuropathic pain identified isolated pain syndromes in 7.4% of cases, with immunotherapy improvement in 85.4% and complete remission in 38.2% of treated patients, particularly following second-line therapies such as rituximab and plasma exchange [9^▪^]. However, longitudinal studies suggest that neuropathic pain may persist despite immunotherapy in some patients, contributing substantially to long-term disability and reduced quality of life [10].

Experimental studies now provide substantial mechanistic support linking CASPR2-Ab to neuropathic pain. Early pathogenic studies demonstrated that the passive transfer of patient-derived CASPR2-Ab induces mechanical hypersensitivity in mice without overt neural injury, supporting a direct functional effect on sensory neurons rather than secondary inflammatory damage [3]. CASPR2 is known to organise Kv1-channel complexes at juxtaparanodal regions of myelinated axons and regulates Kv1-channel localisation in dorsal root ganglion (DRG) neurons [3_,_^14^^▪▪^]. In vitro studies further showed that CASPR2-Ab binds to mouse DRG neurons, reduces membrane-associated Kv1-channel expression, and increases sensory neuronal excitability [3]. More recent work demonstrated that CASPR2-Ab disrupts interactions between CASPR2 and contactin-2 and alters the organisation of Kv1-channel complexes, impairing coordinated electrical signalling [14^▪▪^_,_^15^]. At the molecular level, pain-associated sera increase spatial separation between CASPR2 and Kv1 channels along sensory axons and reduce Kv-channel conductance, while monoclonal CASPR2-Ab induces sensory neuronal hyperexcitability in rodent models [14^▪▪^_,_^15^]. Recent clinical-mechanistic studies further support that CASPR2-associated pain reflects broader nociceptive sensitisation extending beyond classical neuropathic pain [13].

In contrast to CASPR2 autoimmunity, LGI1-associated pain more commonly presents as a diffuse sensory syndrome involving neuropathic pain, dysautonomia, and small-fibre dysfunction, sometimes occurring in the absence of overt limbic encephalitis [7_,_^16^].

While limited mechanistic work has directly investigated the pain-inducing pathogenicity of LGI1-Ab, recent work finds this important regulator of neuronal excitability to be highly expressed in DRG and spinal neurons [17]. Conditional LGI1 deletion in mice produces mechanical hypersensitivity, dorsal horn hyperexcitability, enhanced spinal wind-up, and increased DRG neuronal excitability [17]. This increased excitability is associated with reduced Kv1 currents, consistent with mechanistic studies showing that LGI1-Ab disrupts Kv1-channel organisation and alters neuronal firing properties in central nervous system (CNS) neurons [17_,_^18^]. Importantly, pain frequently improves following immunotherapy, supporting a predominantly functional rather than destructive mechanism [7_,_^16^].

## SMALL-FIBRE NEUROPATHY

Small-fibre neuropathy (SFN), a disorder affecting thinly myelinated Aδ and unmyelinated C-fibres, is recognised to include immune-mediated subgroups characterised by neuropathic pain, autonomic dysfunction, and sensory hyperexcitability [19_,_^20^]. Autoantibodies directed against plexin-D1, fibroblast growth factor receptor 3 (FGFR3), trisulphated heparin disaccharide (TS-HDS), and, more recently, MX dynamin-like GTPase 1 (MX1) have been identified in subsets of patients with cryptogenic SFN [21,22,23^▪^]. Clinically, these seropositive phenotypes may extend beyond classical length-dependent SFN and can include non-length-dependent sensory neuronopathy or broader sensory-predominant polyneuropathy syndromes [5^▪^^▪^,^24^]. Clinical studies suggest immunotherapy responsiveness in selected seropositive SFN cohorts, particularly in plexin-D1-associated disease, although controlled IVIG studies in TS-HDS/FGFR3-associated SFN have yielded negative or inconclusive results [25_,_^26^]. Experimental studies across rodent, cellular, and human tissue models increasingly support altered DRG excitability and sensory neuronal dysfunction as potential contributors to pain in immune-mediated SFN, although the precise pathogenic role of several autoantibodies remains uncertain [5^▪^^▪^,^21^,^23^^▪^,^24^].

A more direct nociceptor-targeting mechanism has been described for plexin-D1-Ab. These antibodies selectively bind unmyelinated pain-conducting DRG neurons and have been identified across a spectrum of chronic neuropathic pain syndromes, including SFN and painful trigeminal neuropathy [21_,_^27^]. Consistent with direct pathogenicity, passive transfer of patient IgG induces mechanical and thermal hypersensitivity in mice, together with increased phosphorylated ERK expression in small DRG neurons, effects abolished by preabsorption with recombinant plexin-D1 [21]. Additional studies suggest that plexin-D1-associated neuropathic pain may involve dysfunction of small sensory neurons and altered DRG neuron signalling, potentially related to the known role of semaphorin–plexin pathways in sensory axonal organisation and neuronal guidance [21]. These findings support a direct pathogenic role for plexin-D1 autoantibodies in nociceptive sensory neurons.

More recent work has suggested that FGFR3 autoantibodies may also alter sensory neuronal function. FGFR3-seropositive patients demonstrate neuropathic pain phenotypes extending beyond classical SFN and are suggestive of DRG dysfunction, while FGFR3-positive patient sera increase DRG neuron excitability, most likely through activation of ERK and p38 MAPK signalling pathways, and induce mechanical hypersensitivity in mice [5^▪▪^]. However, whether DRG neurons represent the primary pathogenic target remains uncertain, as recent transcriptomic and protein-expression studies demonstrate minimal FGFR3 expression in human DRG and peripheral nerve tissue [24].

Additional antibody targets may exist in SFN, including MX1, an interferon-induced protein implicated in neuronal excitability and ion channel regulation, such as TRPC6 [23^▪^]. Patients with autoimmune SFN showed elevated anti-MX1-Ab, together with IgG binding to unmyelinated peripheral nerve fibres [23^▪^]. Although MX1 may regulate DRG neuronal excitability, since its overexpression depolarises DRG neurons and increases action potential firing, anti-MX1 IgG itself did not alter neuronal excitability, suggesting a more indirect contribution through broader immune-mediated or inflammatory mechanisms [23^▪^].

## FIBROMYALGIA

A current focus of research is the study of autoantibody involvement in FMS, a chronic nociplastic pain disorder characterised by widespread musculoskeletal pain, fatigue, sleep disturbance, cognitive dysfunction, and sensory hypersensitivity [28]. Although no single dominant antigen has been identified, passive transfer studies show that FMS-IgG induces mechanical and cold hypersensitivity, nociceptor hyperactivity, and small-fibre pathology in rodents, supporting a pathogenic role for circulating autoantibodies [4_,_^29^_,_^30^].

Regarding antigenic targets, FMS patient-IgG demonstrates increased binding to live murine cultured satellite glial cells (SGCs) and fixed human DRG tissue sections, with elevated anti-SGC IgG levels correlating with greater pain intensity, higher FMS impact scores, and increased pressure sensitivity in subsets of patients [4_,_^29^_,_^31^^▪^]. More detailed immunophenotyping using fixed rat DRG sections identified heterogeneous IgG binding patterns involving FABP7-positive SGC, TRPV1-positive nociceptive neurons, and other neuronal subpopulations [31^▪^]. These findings support immunologically distinct FMS subgroups associated with differing sensory phenotypes [31^▪^]. However, anti-SGC autoreactivity alone does not appear sufficient to confer pathogenicity, as post-acute COVID syndrome demonstrates similarly increased anti-SGC IgG binding to murine SGC cultures without reproducing pronociceptive effects in passive-transfer models [30]. Furthermore, antibody involvement is likely not a ubiquitous feature across FMS. For example, 63% of patients in one recent cohort showed no IgG binding to pain-relevant DRG tissue [31^▪^], while other studies suggest that non-antibody immune mechanisms, including pronociceptive neutrophil activity, may also contribute to FMS-related pain [32]. More recently, and in addition to actions on nociceptors, in mouse passive-transfer models, FMS-IgG sensitises large-diameter Aβ mechanoreceptors to mechanical stimulation and induces de novo cold sensitivity, consistent with human microneurography findings [6]. In addition, ex vivo skin–saphenous nerve recordings from mice treated with FMS-IgG demonstrate persistent after-discharge in mechano-sensitive afferents following stimulation, suggesting impaired signal termination and prolonged sensory encoding rather than simple stimulus-evoked hyperexcitability [33].

Mast-cell activation has also emerged as a possible effector mechanism. In mouse passive-transfer studies, FMS-IgG promotes MRGPRX2/Mrgprb2-dependent mast-cell recruitment and IL-6 secretion, while genetic deletion or ablation of mast cells abolishes IgG-induced mechanical and cold hypersensitivity in mice [34]. Human FMS skin biopsies similarly demonstrate increased mast-cell density and enhanced tryptase staining, supporting the clinical relevance of mast-cell activation [34].

## INJURY-ASSOCIATED AUTOIMMUNITY

Tissue injury provides an important context in which autoreactive antibodies can become pronociceptive. Structural disruption and local inflammation may create permissive neuroimmune environments that allow autoantibodies to enhance nociceptive signalling and promote persistent pain.

For example, following chronic constriction injury (CCI) or sciatic nerve crush (NC) in mice, IgG accumulates within injured nerves, DRG, and spinal cord tissue, while B-cell depletion prevents the development of mechanical allodynia [35^▪^,^36^^▪^]. Passive transfer studies further demonstrated that injury-associated IgG is itself pronociceptive [36^▪^]. However, these effects differ between injury types. Although autoreactive IgG and immune complexes also increase after spared nerve injury (SNI) and spinal nerve ligation (SNL), B-cell depletion does not prevent allodynia in these models [36^▪^]. Consistent with this, the expression of the Fc receptor γ subunit (FcRγ) increases after CCI and NC but not after SNI or SNL, suggesting that injury-specific regulation of Fc receptor signalling determines whether autoreactive antibodies become pathogenic [36^▪^].

A similar injury-dependent mechanism has been described in complex regional pain syndrome (CRPS). Passive transfer of IgG from patients with persistent CRPS produces hyperalgesia and oedema only in the presence of prior tissue injury, supporting the concept that circulating autoantibodies become pathogenic within an injury-primed sensory environment [37_,_^38^]. In addition to actions on nociceptors [39], CRPS-IgG induces marked spinal microglial and astrocyte activation, together with increased microglial IL-1β production, while pharmacological IL-1 receptor blockade reverses established sensitisation, also implicating central neuroimmune amplification downstream of injury-triggered autoimmunity [37].

Related mechanisms have also been identified in chronic musculoskeletal pain states. In lumbar disc puncture and osteoarthritis models, mice lacking mature B cells are protected from chronic allodynia and hyperalgesia, while passive transfer of injury-associated IgM or IgG reproduces sensitisation in recipient animals [40_,_^41^]. These models additionally demonstrate immune-complex deposition, complement activation, and increased Fcγ receptor signalling within injured tissues and corresponding spinal pathways [40–42]. Importantly, antibodies generated after one form of tissue injury fail to reproduce sensitisation in unrelated injury models, suggesting that distinct injuries generate unique combinations of neoantigens and autoreactive antibodies [40].

Recent work further implicates neonatal Fc receptor (FcRn) signalling in sustaining antibody-mediated pain. FcRn blockade reduces IgG accumulation within the lumbar DRG and spinal cord after CCI and alleviates established mechanical allodynia, even at chronic stages after injury [35^▪^]. Conversely, rheumatoid arthritis demonstrates that not all injury-associated autoantibodies are pronociceptive. A subset of anti-citrullinated protein antibodies instead suppresses arthritis through FCGR2B-dependent anti-inflammatory signalling, illustrating that tissue context, Fc receptor usage, and antigen specificity determine whether autoreactive antibodies exert pathogenic or protective effects [43].

## ANATOMICAL AND MECHANISTIC FEATURES OF AUTOIMMUNE PAIN

Although autoantibody-associated pain disorders differ in clinical phenotype and antigen specificity, several recurring themes emerge across these conditions.

Across multiple conditions, autoantibodies either target antigens expressed by DRG neurons and SGC or demonstrate accumulation within the DRG and anatomically connected sensory structures following passive transfer [3_,_^23^^▪^,^29^_,_^31^^▪^]. The DRG may be particularly susceptible, given that the blood–nerve barrier is most leaky at this level, allowing circulating antibodies access to neuronal, glial, and immune components within sensory ganglia more readily, while DRG neurons themselves represent critical substrates for peripheral sensitisation and chronic pain generation. This relative accessibility may also make the DRG an attractive therapeutic target, as peripherally restricted strategies that reduce pathogenic autoantibodies or interrupt Fc receptor signalling could potentially modulate nociceptive sensitisation without requiring extensive CNS penetration [35^▪^,^36^_,_^42^_,_^44^]. Several studies additionally demonstrate direct functional effects on sensory neuronal excitability, including altered ion-channel organisation, increased spontaneous firing, and persistent post-stimulus activity [3_,_^5^_,_^33^]. Although the DRG emerges as a central site of convergence, autoimmune pain mechanisms are not restricted to sensory ganglia alone. In vitro and ex vivo studies demonstrate that some autoantibodies can also bind peripheral sensory axons and specialised nodal or juxtaparanodal structures when accessible, while injury-associated models further implicate the spinal dorsal horn and neuroimmune circuits in sustaining nociceptive sensitisation [14^▪^,^36^_,_^37^].

In some conditions, antibodies directly disrupt ion-channel organisation or receptor-mediated signalling within sensory neurons, whereas others primarily involve glial activation, inflammatory amplification, or Fc receptor-dependent neuroimmune pathways [3_,_^5^_,_^21^_,_^44^]. Consequently, autoimmune pain does not rely solely on direct increased neuronal excitability but additionally may involve abnormal afferent recruitment, persistent post-stimulus activity, inflammatory amplification, and impaired coordination of nociceptive signalling [6_,_^17^_,_^33^_,_^37^]. These disorders, therefore, demonstrate substantial mechanistic heterogeneity, which may contribute to the marked variability in sensory phenotype, chronicity, and treatment responsiveness observed across autoimmune pain conditions. For example, CASPR2- and LGI1-associated pain predominantly reflects direct effects on sensory neuronal excitability and may be particularly responsive to therapies that reduce pathogenic autoantibodies. By contrast, FMS and injury-associated models additionally implicate glial, mast-cell, and Fc receptor-dependent mechanisms, suggesting that downstream inflammatory and neuroimmune pathways may represent important complementary therapeutic targets [3_,_^14^^▪^,^17^_,_^34^_,_^35^_,_^44^]. Such mechanistic differences may also influence whether autoantibodies primarily drive pain initiation or contribute to its long-term maintenance. Across several passive-transfer models, patient-derived autoantibodies are sufficient to induce nociceptive sensitisation and pain-related behaviours [3_,_^21^_,_^29^_,_^37^], suggesting an important role in pain initiation, where induced pain can normalise once antibody exposure is stopped [30]. However, their role appears to extend beyond initiation, as reducing autoreactive IgG can also reverse established pain, albeit less effectively than when IgG is reduced at earlier stages after pain induction [35^▪^], suggesting that secondary neuronal and neuroimmune changes can become established over time, consistent with continuing pain in some patients despite immunotherapy [3_,_^10^_,_^29^_,_^37^].

One potentially important but still incompletely characterised factor that warrants further investigation in autoimmune pain disorders is IgG subclass-specific effector function. In neurofascin-associated neuropathies, for example, different IgG subclasses are associated with distinct patterns of complement activation and nodo-paranodal damage, highlighting the potential value of subclass-based disease stratification [45]. In CASPR2-associated disease, IgG4 autoantibodies predominantly disrupt protein interactions within the VGKC without complement activation, whereas non-IgG4 subclasses are associated with inflammatory and complement-mediated injury [14_,_^45^]. However, clinical studies demonstrate that subclass alone does not reliably predict pain phenotype or severity, indicating that additional factors influence pathogenicity [13].

Context dependence represents another major recurring principle. In CRPS and injury-associated models, tissue injury and local immune activation are required for antibody-mediated sensitisation [36_,_^37^]. In FMS, anti-SGC reactivity is not uniformly pronociceptive, demonstrating that antibody binding is not necessarily sufficient to determine pain expression and that further contextual factors likely impact pathogenicity [30].

## CLINICAL IMPLICATIONS AND CONCLUSION

Although accumulating evidence supports a role for autoantibodies in selected chronic pain disorders, important uncertainties remain. Experimental models have been invaluable for establishing pathogenic mechanisms, demonstrating that patient-derived autoantibodies can induce pain-related behaviours and nociceptive sensitisation following passive transfer [3_,_^21^_,_^29^_,_^37^]. However, these paradigms typically involve short-term exposure to purified IgG or pooled patient sera and may not fully recapitulate key features of human disease, including chronic antibody exposure, tissue injury, and evolving neuroimmune interactions. For example, passive-transfer models of FMS and CASPR2 autoimmunity demonstrate antibody-induced sensitisation but do not reproduce the prolonged disease course observed in patients, while injury-dependent mechanisms implicated in CRPS and nerve injury models may require additional inflammatory or tissue-derived signals that are not fully captured by antibody transfer alone [3_,_^29^_,_^36^_,_^37^]. Clinical evidence is also uneven across conditions, with relatively few controlled interventional studies available, and much of the current literature relies on observational cohorts [13_,_^25^_,_^26^_,_^31^^▪^]. Furthermore, the presence of autoantibodies does not necessarily establish pathogenicity, and the diagnostic performance and clinical utility of several emerging autoantibody assays remain incompletely defined [19_,_^20^_,_^22^_,_^24^_,_^31^^▪^].

Despite these limitations, recognition of autoantibody-mediated pain mechanisms reframes certain forms of chronic pain as potentially autoimmune-driven. Clinical improvement following immunotherapy suggests that at least some chronic pain states are therapeutically modifiable through immune-directed approaches [7_,_^8^_,_^16^].

These observations identify several therapeutic entry points, including the reduction of circulating autoantibodies, targeting antibody-producing B cells, limiting IgG recycling through neonatal FcRn blockade, and interrupting downstream Fc receptor, complement, or cytokine signalling pathways [35_,_^37^,^40^–^42^]. Experimental studies additionally suggest that targeting glial activation, IL-1 signalling, mast-cell pathways, or complement activation may reduce sensitisation even after pain becomes established [34_,_^37^_,_^41^].

Nevertheless, variable therapeutic responsiveness remains an important challenge. Pain may persist despite immunotherapy, even when other neurological manifestations improve, suggesting that autoantibodies may initiate nociceptive sensitisation that later becomes sustained by secondary peripheral or central neuroplastic changes [10_,_^13^_,_^30^_,_^43^_,_^46^]. Variable treatment responses additionally suggest that factors such as antibody subclass, tissue accessibility, chronicity of disease, and the extent of established neuroimmune or central sensitisation may influence the reversibility of pain [10_,_^13^_,_^14^_,_^30^_,_^45^].

Overall, the expanding field of autoantibody-mediated pain suggests that autoantibodies may provide a mechanistic explanation for at least a subset of chronic pain conditions and highlights the need for mechanism-based approaches integrating autoantibody profiling, neuroimmune mechanisms, and clinical phenotyping to improve patient stratification and support the development of more targeted therapies.
